# Ship Type Classification by Convolutional Neural Networks with Auditory-Like Mechanisms

**DOI:** 10.3390/s20010253

**Published:** 2020-01-01

**Authors:** Sheng Shen, Honghui Yang, Xiaohui Yao, Junhao Li, Guanghui Xu, Meiping Sheng

**Affiliations:** School of Marine Science and Technology, Northwestern Polytechnical University, Xi’an 710072, China;shensheng@mail.nwpu.edu.cn (S.S.); yaoxiaohui@mail.nwpu.edu.cn (X.Y.); ljhhjl@mail.nwpu.edu.cn (J.L.); hsugh@mail.nwpu.edu.cn (G.X.); smp@nwpu.edu.cn (M.S.)

**Keywords:** machine learning, neural network, ship radiated noise, underwater acoustics

## Abstract

Ship type classification with radiated noise helps monitor the noise of shipping around the hydrophone deployment site. This paper introduces a convolutional neural network with several auditory-like mechanisms for ship type classification. The proposed model mainly includes a cochlea model and an auditory center model. In cochlea model, acoustic signal decomposition at basement membrane is implemented by time convolutional layer with auditory filters and dilated convolutions. The transformation of neural patterns at hair cells is modeled by a time frequency conversion layer to extract auditory features. In the auditory center model, auditory features are first selectively emphasized in a supervised manner. Then, spectro-temporal patterns are extracted by deep architecture with multistage auditory mechanisms. The whole model is optimized with an objective function of ship type classification to form the plasticity of the auditory system. The contributions compared with an auditory inspired convolutional neural network include the improvements in dilated convolutions, deep architecture and target layer. The proposed model can extract auditory features from a raw hydrophone signal and identify types of ships under different working conditions. The model achieved a classification accuracy of 87.2% on four ship types and ocean background noise.

## 1. Introduction

The soundscape of the oceans is heavily affected by human activities, especially in coastal waters. The increase of the noise level in oceans is correlated with burgeoning global trade with the expansion of shipping. Automatic recognition of ship type by ship radiated noise is not only affected by the complicated mechanism of noise generation, but is also affected by the complex underwater sound propagation channel. The conventional recognition methods based on machine learning generally include three stages—feature extraction, feature selection and classifier design.

The conventional feature extraction methods for ship radiated noise include waveform features [1,2], auditory features [3,4], wavelet features [5] and so on. These manually designed features are limited in their ability to capture variations in complex ocean environments and ship operative conditions for the use of fixed parameters or filters. Biophysical based models [3,4] are limited to early auditory stages for extracting auditory features. Auditory features designed from perceptual evidence always focus on the properties of signal description rather than the classification purpose [6]. These features do not utilize the plastic mechanism and representation at various auditory stages to improve the recognition performance. Although the noise features or redundant features can be removed by feature selection methods [7], the inherent problem of manually designed features still cannot be solved radically.

Support vector machine (SVM) was used to recognize ship noise using manually designed features [7]. With the increase of data size, hierarchical architectures have been shown to outperform shallow models. Spectrogram [8], probabilistic linear discriminant analysis and i-vectors [9] were used as the input of neural classifiers to detect ship presence and classify ship types. For the application of deep learning, Kamal [10] used a deep belief network and Cao [11] used a stacked autoencoder. A competitive learning mechanism [12,13] was used to increase cluster performance during the training of the deep network. In these works, classifier design and feature extraction were separated from each other. This has a drawback that the designed features may not be appropriate for the classification model.

Deep learning has made it possible to model the original signal as well as to predict targets in a whole model [6,14], to which the auditory system is thought to be adapted. The time convolutional layer in an auditory inspired convolutional neural network (CNN) [6] provided a new way for modeling underwater acoustic signals. However, it did not have enough depth to build an appropriate model to match the expanding acquired dataset. Moreover, the conventional convolutional layer and the fully connected layer led to numerous parameters.

In this paper, we present a deep architecture with the aim of capturing the functionality and robustness of the auditory system to improve recognition performance. The key element in the approach is a deep architecture with time and frequency-tolerant feature detectors, which are inspired by neural cells along the auditory pathway. The early stage auditory model is derived from the auditory inspired CNN, in which the time convolutional layer is improved by dilated convolution. The construction of the deep network refers to inception and residual network [15] in the field of machine vision, in which some ideas are also found in the auditory pathway. Thus, the frequency convolutional layers in Reference [6] are improved to increase the depth of the network. At the final stage, the substitution of the fully connected layer by global average pooling at target layer greatly reduces the parameters. The main findings of this paper are briefly summarized as follows:

- The proposed convolutional neural network could transform the time domain signal into a frequency domain that is similar to gammatone spectrogram.
- Deep architecture of a neural network derived from an auditory pathway improves the classification performance of ship types.
- Auditory filters in convolutional kernals are adaptive in shape during the optimization of the network with the ship type classification task.
- The classification results of the model are robust to ship operative conditions. The increase of distance between ships to hydrophone has a negative effect on recognition results in most cases.

This paper is organized as follows. Section 2 gives an overview of auditory mechanisms and the structure of the proposed model. Section 3 describes details of the model, which include the cochlea model for ship radiated noise modeling and the multistage auditory center model for features learning and targets classification. Section 4 includes experimental data description, experimental setup and results. An overall discussion and directions for future work are concluded in Section 5.

## 2. Model

### 2.1. Auditory Mechanisms

Decades of physiological and psychoacoustical studies [16,17,18] have revealed elegant strategies at various stages of the mammalian auditory system for representation of acoustic signals. Sound is first analyzed in terms of relatively few perceptually significant attributes, followed by higher level integrative processes.

When sound arrives at the ears, the vibration of the eardrum caused by the sound wave is transmitted to the cochlea in the inner ear via ossicular chain in the middle ear. The cochlea performs two fundamental functions. First, through the vibration of different parts of the basement membrane, the cochlea effectively separates the frequency components of sound. The second function of the cochlea is to transform these vibrations into neural patterns with the help of hair cells distributed along the cochlea [18].

The auditory center is one of the longest central pathways in the sensory system. The acoustic spectrum is extracted early in the auditory pathway at the cochlear nucleus, the first stage beyond the auditory nerve. Multiple pathways emerge from the cochlear nucleus up through the midbrain and thalamus to the auditory cortex. Each pathway passes through different neural structures and repeatedly converges onto and diverges from other pathways along the way [18]. The complexity structure extracts rich and varied auditory percepts from the sound to be later interpreted by the brain. Neurons in the primary cortex have been shown to be sensitive to specific spectro-temporal patterns in sounds [19].

As a result of auditory experience, the systematic long-term changes in the responses of neurons to sound are defined as plasticity. Plasticity and learning probably occurs at all stages of the auditory system [20].

By reviewing the process of auditory perception, we can conclude the following four mechanisms of early and higher auditory stages that are useful for establishing an auditory computational model.

- Auditory processing is hierarchical.
- Neurons throughout the auditory pathway are always tuned to frequency.
- Auditory pathways have different neural structures.
- The auditory system has plasticity and learning properties.

### 2.2. Model Structure

**Figure 1:**
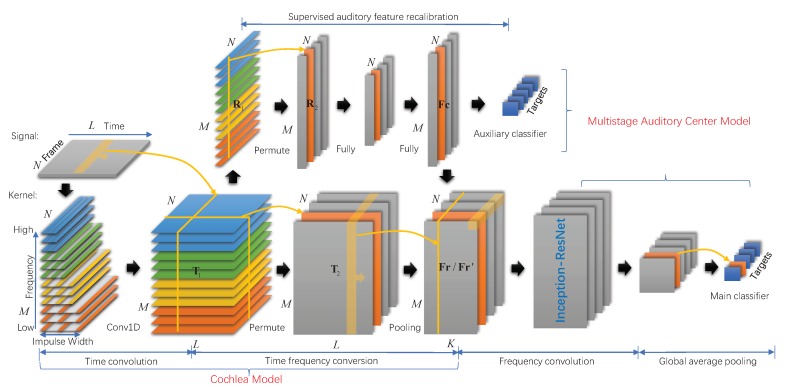
Model structure. The model mainly includes cochlea model and auditory center model. In the time convolutional layer, four colors represent four groups. Dilated convolutions are represented by parallel lines at equal intervals. The time frequency conversion layer includes permute layer and max-pooling layer. At the top of the graph, auditory feature recalibration is implemented by global max-pooling and fully connected layers. Frequency convolutional layers are performed by Inception-ResNet. The final stage includes global average pooling and softmax layer.

The nature of the auditory computational model is to transform the raw acoustical signal into representations that are useful for auditory tasks. In this paper, mechanisms of auditory system are established mathematically in deep CNN for ship type classification. The model mainly includes the cochlea model and the auditory center model. A complete description of the specifications of the network is given in Figure 1.

Based on the research foundation of the human cochlea, a series of multiscale gammatone auditory filters [21] are used to initial the time convolutional layer with dilated convolutions [22]. Ship noise signals are decomposed by convolution operation with auditory filters. Inspired by the function of hair cells, the time frequency conversion layer transforms these decomposed components into amplitudes of its corresponding frequency components—or its frequency spectrum [18]. We introduce an auxiliary classifier with the goal of enhancing the gradient and recalibrating the learned spectrum in supervised manner. Learned spectra are further extracted by deep architecture with inception structures and residual connections [15] to model the multistage auditory pathway. These layers are defined as frequency convolutional layers. The resulting feature maps of the last frequency convolutional layer are fed into a global average pooling layer [23]. Then ship types are predicted in the softmax layer. During the training of the network, auditory filters and features are subject to classification tasks on the basis of matching human auditory systems.

## 3. Methodology

### 3.1. Cochlea Model for Ship Radiated Noise Modeling

The cochlea model is the first stage of the proposed model, it includes the time domain signal decomposition of basement membrane and the time frequency conversion of hair cells. The cochlea model creates a frequency-organized axis known as the tonotopic axis of cochlea.

#### 3.1.1. Time Convolutional Layer with Dilated Auditory Filters

Much is known about the representation of spectral profile in the cochlea [18,21]. The physiologically derived gammatone filter g(t) is shown in (1).

(1) $$g\left(t\right)=at^{n-1}e^{-2\pi bt}cos\left(2\pi ft+\phi \right),$$

where *a* is amplitude, *t* is time in second, *n* is filter’s order, *b* is bandwidth in Hz, *f* is center frequency in Hz, and ϕ is phase of the carrier in radians. Center frequency *f* and bandwidth *b* are set by an equivalent rectangular bandwidth (ERB) [24] cochlea model in (2) and (3).

(2) ERB(f)=24.7(4.37f/1000+1)

(3) b=1.019×ERB(f).

Convolutional kernels of the time convolutional layer represent a population of auditory nerve spikes. A series of gammatone filters of different sizes are used to initialize weight vectors of this layer which forms the primary feature extraction base of the network. This layer performs convolutions over a raw time domain waveform. Suppose the input signal $S(S\in R^{L\times N})$ has *N* frames, each frame length is *L*. As shown in (4), signal S is convolved with kernel $k_{m}$ and added to an additive bias $b_{m}$. Then, the output puts through activation function *f* to form the output feature map $t_{m}\left(t_{m}\in R^{L\times N}\right)$.

(4) $$t_{m}=f\left(S*k_{m}+b_{m}\right),m\in 1,2,\ldots ,M.$$

For time convolutional layer that has *M* kernels, the output $T_{1}=\left[t_{1},t_{2},\ldots ,t_{M}\right]$ will be obtained. We use 128 gammatone filters with center frequencies ranging from 20 to 8000 Hz. For 16 kHz sampling frequency, the impulse widths range from 50 to 800 points approximately. These filters are divided into 4 groups by quartering according to impulse widths. The convolutional kernel widths of the 4 groups are 100, 200, 400, 800 respectively. Thus, the number of parameters is reduced from 128×800 to 32×(100+200+400+800).

**Figure 2:**
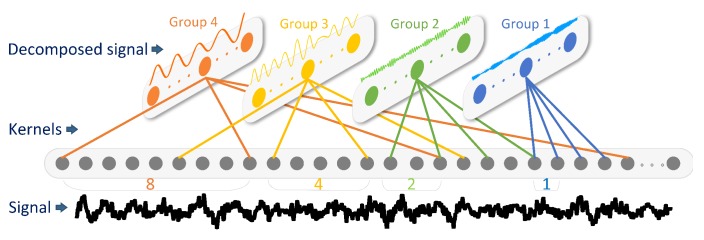
Signal decomposition of time convolutional layer. Black line at the bottom is the input signal. Four colors (orange, yellow, green and blue) at the upper and middle parts represent the four groups. Four capsules at the top represent the outputs of 4 groups. Four colors of straight lines at the middle part represent dilated convolutional kernels with a dilated rate of 8, 4, 2 and 1.

Bigger kernel size means more parameters, which make the network more prone to overfitting. Dilated convolutions could reduce network parameters, while the receptive fields, center frequencies and band widths remain unchanged. To give the 4 groups an equal number of parameters, dilation rates are 1, 2, 4, and 8 respectively for the 4 groups. The number of parameters in this layer are further reduced to 128×100. Figure 2 illustrates the signal decomposition by using underwater noise radiated from a passenger ship. During the recording period, the ship was 1.95 km away from the hydrophone and its navigational speed was 18.4 kn.

#### 3.1.2. Time Frequency Conversion Layer

Stronger vibrations of basement membrane lead to more vigorous neural responses, which are further transformed by hair cells. The amplitude of the decomposed signal can be regarded as a neural response or frequency spectrum. The proposed time frequency conversion layer transforms the output of the time convolutional layer into a frequency domain. This is accomplished by a permute layer and a max-pooling layer. As shown in (5), $T_{1}$ is permuted to $T_{2}=\left[\tau_{1},\tau_{2},\ldots ,\tau_{N}\right]$, where $\tau_{n}\in R^{L\times M},n=1,2,\ldots ,N$.

(5) $$T_{2}=permute\left(T_{1}\right).$$

As shown in (6), the amplitude of $\tau_{n}$ within regular time bins is calculated as ${\mathrm{fr}}_{n}\left(n=1,2,\ldots N,{\mathrm{fr}}_{n}\in R^{K\times M}\right)$ by max-pooling along time axis. The output of this layer is $\mathrm{Fr}=[{\mathrm{fr}}_{1},{\mathrm{fr}}_{2},\ldots ,{\mathrm{fr}}_{N}]$. Thus, the internal representation of sound is calculated as a spectro-temporal excitation pattern which provides a clearer picture of sound.

(6) $${\mathrm{fr}}_{n}=max-pooling\left(\tau_{n}\right).$$

### 3.2. Multistage Auditory Center Model for Feature Extraction and Classification

The output of the time convolutional layer is divided into two routes. One route is the time frequency conversion layer, then directly through the deep neural network to model the multistage auditory pathway. Another route performs auditory feature recalibration.

#### 3.2.1. Supervised Auditory Feature Recalibration

Given the relatively large depth of the network, the ability to propagate gradients back through all the layers should be enhanced, especially for time convolutional layer at the front of the whole network. Therefore, we propose an auditory feature recalibrate block on the basis of the recalibrate block [25]. This block takes $T_{1}$ as the input. As shown in (7), global max pooling is used to aggregate $t_{m}$ across frame length.

(7) $$r_{m}=max-pooling\left(t_{m}\right),m=1,2,\ldots ,M.$$

The output $r_{m}\left(r_{m}\in R^{N}\right)$ is the amplitude of all the frames in $t_{m}$. The output of this layer is $R_{1}=\left[r_{1},r_{2},\ldots ,r_{M}\right]$. It is permuted to $R_{2}=\left[\gamma_{1},\gamma_{2},\ldots ,\gamma_{N}\right]$, where $\gamma_{n}\in R^{M},n=1,2,\ldots ,N$. Then, it is followed with two fully connected layers to capture the dependencies of frequency components. The activation function of the fully connected layers are Rectified Linear Unit (ReLU) and sigmoid, respectively. The equation of the two layers are shown in (8):

(8) $${\mathrm{fc}}_{n}=sigmoid\left(\upsilon_{n}ReLU\left(\omega_{n}\gamma_{n}\right)\right),n=1,2,\ldots ,N.$$

$\mathrm{Fc}=[{\mathrm{fc}}_{1},{\mathrm{fc}}_{2},\ldots ,{\mathrm{fc}}_{N}]$ is the output of the fully connected layers, where ${\mathrm{fc}}_{n}\left({\mathrm{fc}}_{n}\in R^{M}\right)$ corresponds to the $n^{th}$ frame. $W=[\omega_{1},\omega_{2},\ldots ,\omega_{N}]$ and $U=[\upsilon_{1},\upsilon_{2},\ldots ,\upsilon_{N}]$ are weight vectors of the two layers. Fc is also divided into two routes. One route is fed directly into a softmax layer to make an auxiliary classifier. We would expect to increase the gradient signal by adding the loss of the auxiliary classifier to the total loss of the network. Another route is the auditory feature recalibration shown in (9).

(9) $${\mathrm{fr}}_{n}^{\prime }={\mathrm{fc}}_{n}\times {\mathrm{fr}}_{n},n=1,2,\ldots ,N,$$

where ${\mathrm{fr}}_{n}^{\prime }\left({\mathrm{fr}}_{n}^{\prime }\in R^{K\times M}\right)$ is channel-wise multiplication between ${\mathrm{fc}}_{n}$ and ${\mathrm{fr}}_{n}$. The output of the layer is ${\mathrm{Fr}}^{\prime }=\left[{\mathrm{fr}}_{1}^{\prime },{\mathrm{fr}}_{2}^{\prime },\ldots ,{\mathrm{fr}}_{N}^{\prime }\right]\left({\mathrm{Fr}}^{\prime }\in R^{K\times M\times N}\right)$. This operation can be interpreted as a means of selecting the most informative frequency components of a signal in supervised manner. The recalibrated auditory features could establish the correlation between features and categories.

#### 3.2.2. Deep Architecture for Feature Learning

Auditory perception depends on the integration of many neurons along the multistage auditory pathway. These neurons likely facilitate frequency topological topographic maps of most hearing region [26]. The proposed frequency convolutional layers perform convolution in both frequency and time axis to extract spectro-temporal patterns embedded in a ship radiated noise signal.

However, a drawback of a deep network constructed with a standard convolutional layer is the dramatically increased use of computational resources. Inception-Resnet [15] has been shown to achieve very good performance in image recognition at a relatively low computational cost. In this paper, a deep neural network with inception structures and residual connections are introduced in frequency convolutional layer to perform the auditory task. Multiscale convolutional kernels in inception block can be interpreted as simulating the different neural structures of auditory pathways. Inception structures have the ability to learn spectro-temporal patterns of different scales with less parameters. Residual connections can be interpreted as simulating the convergence and divergence between different pathways. The architecture allows for increasing the number of layers and units to form the multistage of auditory system. These layers could also preserve locality and reduce spectral variations of the line spectrum in ship radiated noise.

We use an global average pooling layer [23] at the final stage to generate one feature map for each ship type. The resulting vector is fed directly into softmax layer to predict targets. Compared with fully connected layers, global average pooling layer is more native to the convolution structure by enforcing correspondences between feature maps and categories. Moreover, there is no parameter to optimize in global average pooling layer thus overfitting is avoided at this layer.

## 4. Experiment

### 4.1. Experimental Dataset

Our experiments were performed on hydrophone acoustic data acquired by Ocean Networks Canada observatory. The data were measured using an Ocean Sonics icListen AF hydrophone placed at 144 m–147 m below sea level. Ship radiated noise data were from ships in a 2 km radius while no other ships were present in a 3 km radius. The duration of the recordings vary from about 5 to 20 min, depending on navigational speed and position. Each recording was sliced into several segments to make up the input of neural network. Each sample was a segment of 3 s duration and was divided into short frames of 1 s. Acoustic data were resampled to a sampling frequency of 16 kHz. Classification experiments were performed on ocean background noise and four ship types (Cargo, Passenger ship, Tanker, and Tug). The four ship type categories were designated by the World Shipping Encyclopedia from Lloyd’s Registry of Ships. About 29 months of data were collected, the first 18 months for training and the remaining 11 months for testing. The dataset comprised about 140,000 training samples (771 recordings) and 82,000 validation samples (449 recordings).

### 4.2. Classification Experiment

To evaluate the classification performance of the proposed model, we report the classification accuracy against the previous proposed auditory inspired CNN and manually designed features. These hand designed features included waveform features [1,2], Mel-frequency Cepstral Coefficients (MFCC) [3], wavelet features [5], auditory features [7] and spectral [11,27]. Waveform features included peak-to-peak amplitude features, zero-crossing wavelength features and zero-crossing wavelength difference features. MFCC features were extracted based on a linear cosine transform of a log power spectrum on a nonlinear Mel scale of frequency. Auditory features were extracted based on the Bark scale and masking properties of the human auditory system. Wavelet features contained a low frequency envelope of wavelet decomposition and entropy of zero-crossing wavelength distribution density of all levels of wavelet signals. For the calculation of spectral, two pass split window (TPSW) was applied subsequently after a short time fast Fourier transform. Signals were windowed into frames of 256 ms before extracting features. The extracted features on frames were stacked or averaged to feed into support vector machine (SVM), back propagation neural network (BPNN) or CNN to classify ship types. The kernel function of SVM was the radial basis function (RBF). The penalty factor and kernel parameter of RBF were selected by grid search. The SVM ensemble was performed by the AdaBoost algorithm. The used BPNN had one hidden layer with 30 hidden units. The structure of CNN from the bottom up was a convolutional layer with 128 feature maps, a max pooling layer, convolutional layer with 64 feature maps, max pooling layer and fully connected layer with 32 units. Kernel size was 5×5 and pooling size was 2×2. The learning rate was 0.1 and momentum was 0.9. When training the proposed network, optimization was performed using RMSprop with learning rate 0.001, momentum 0.9, and a minibatch size of 50. The results are shown in Table 1. Our experiments demonstrate that the proposed method remarkably outperforms manually designed features. Benefiting from the improvements in the network structure, the accuracy has been greatly improved compared with auditory inspired CNN.

The confusion matrix of the proposed model on test data is shown in Table 2. The accuracy is at the bottom right corner. Both the precision and recall of background noise are higher than all ship types. This result indicates that it is easier to detect ship presence than classify ship types. The confusion between Cargo and Tanker are larger than other categories. This may be because the two categories always have similar propulsion systems, gross tonnage and ship size.

We evaluated the performance of the proposed model on recordings by majority voting. One recording would be classified to a category to which the most samples in the recording are classified. The confusion matrix is shown in Table 3. The obtained accuracy is 94.75% at the bottom right corner. The recall and precision of all categories are improved compared with Table 2. Although individual samples could be misidentified, we can still make a correct recognition results of the whole signal by majority voting.

### 4.3. Operative Conditions Analysis

**Figure 3:**
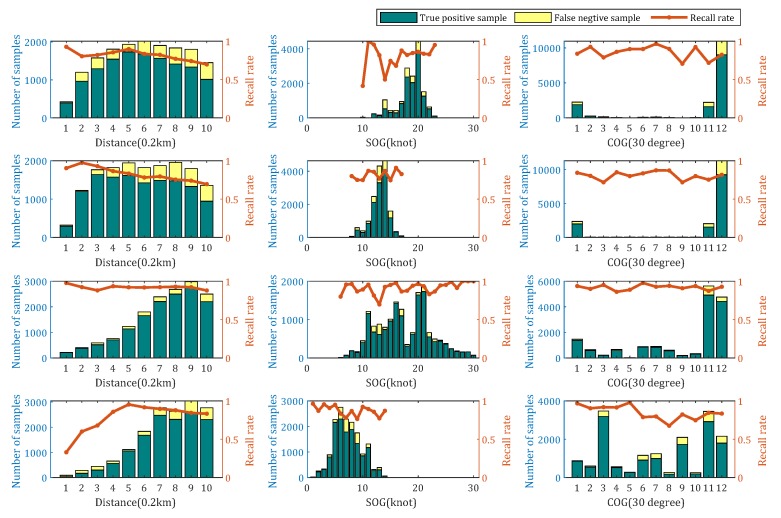
Operative condition and classification results analysis. Four rows from top to bottom represent Cargo, Tanker, Passenger ship and Tug, respectively. Histograms are the number of samples under different operative conditions. Yellow and green histograms represent the false negative samples and true positive samples, respectively. Orange lines are recall rates.

We analyzed the recognition results and operative conditions together in order to observe how the accuracy varies with ship operative conditions. Speed over ground (SOG), course over ground (COG) and distance to hydrophone were analyzed. Different ship types usually had different speeds and routes. Most ships were northbound, whereas only some passenger ships and tugs went in other directions. Because of these differences between ship types, it is necessary to analyse each ship type separately. From Figure 3, we can see that, with the increase of distance between ships and hydrophone, the recall rate of Cargo, Passenger ships and Tanker decreased. It is hard to find obvious laws about the influence of SOG and COG. The results indicate that the proposed model is robust to ship operative conditions. The detection results of passenger ships in each operative condition were obviously better than for the other ship types. This may be because the samples of a passenger ship are uniformly distributed in operative conditions and the classification model can fit it better.

### 4.4. Visualization

#### 4.4.1. Learned Auditory Filter Visualization

**Figure 4:**
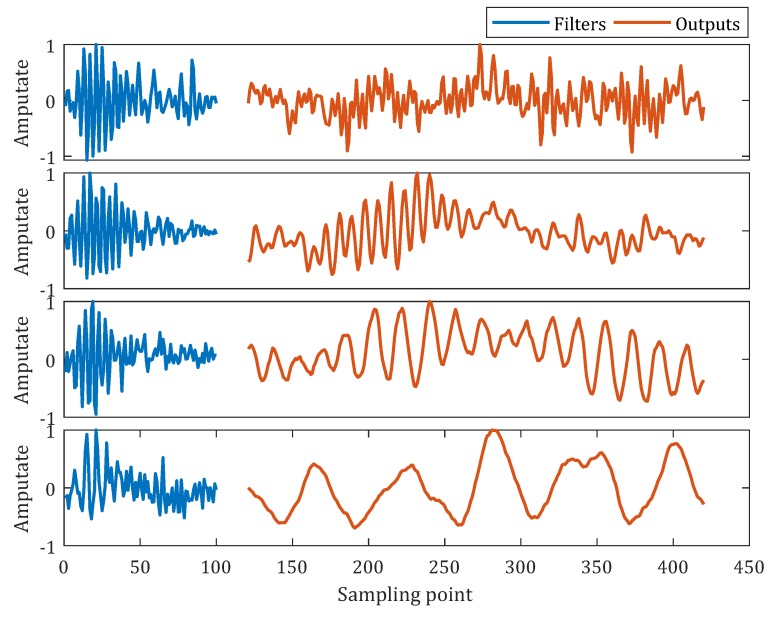
Visualization of learned auditory filters and their outputs in the time convolutional layer. Blue lines are learned auditory filters and orange lines are the corresponding output feature maps. The 4 panels from top to bottom represent the dilation rate of 1, 2, 4, and 8, respectively. For each panel, vertical axis represents the amplitude of filter and decomposed signal and horizontal axis represents the time domain sampling points of the filter and decomposed signal.

To observe learned auditory filters in the time convolutional layer, we selected one convolutional kernel (learned filters) from each of the 4 groups. Output feature maps corresponding to the 4 selected kernels were also extracted. As shown in Figure 4, the training of the network modified the shapes of these filters. The use of dilated convolution enables the 4 groups to have the same kernel width. The output feature maps are decomposed signals whose center frequencies are consistent with the original auditory filters. The dilated convolution can reduce parameters as well as preserve the center frequency of the auditory filter.

#### 4.4.2. Learned Spectrogram Visualization

**Figure 5:**
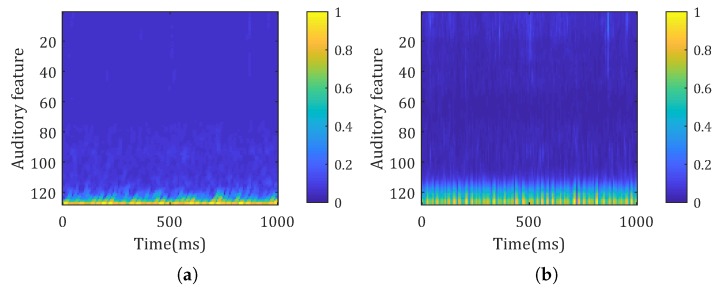
Comparison of learned spectrogram and gammatone spectrogram. (**a**) Learned spectrogram. (**b**) Gammatone spectrogram.

Outputs of the time frequency conversion layer were extracted as a learned spectrogram. It was compared with the gammatone spectrogram in Figure 5. The frame length and hop time for the gammatone spectrogram were the same as the kernel size and strides in the time convolutional layer. Thus, the dimension of the gammatone spectrogram was the same as the learned spectrogram. The learned spectrogram generated by the network is similar to the gammatone spectrogram. The network reserved low frequency components in signal and smoothed noises in high frequency components.

**Figure 6:**
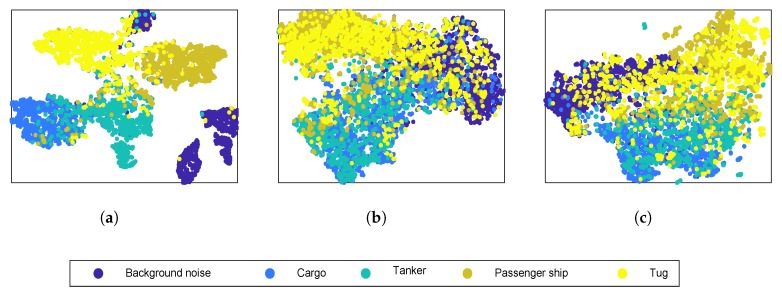
Feature visualization by t-distributed stochastic neighbor embedding (t-SNE). Five thousand samples selected randomly from test data were used to perform the experiments. Dots of different colors represent different types of ships. (**a**) Output of the whole network. (**b**) Learned spectrogram. (**c**) Gammatone spectrogram.

The data visualization method t-distributed stochastic neighbor embedding (t-SNE) [28] was used to visualize extracted features by giving each sample a location in a two dimensional map. In Figure 6, the output of the whole network, learned spectrogram in time frequency conversion layer and gammatone spectrogram were visualized. As shown in Figure 6a, the last layer constructs a map in which most classes are separate from other classes. Figure 6b,c are the samples distribution of learned spectrograms and gammatone spectrograms, respectively. There are larger overlaps between the classes compared with Figure 6a. The samples distribution in Figure 6b is slightly better than that in Figure 6c. The results indicate that the proposed model could provide better insight into class structure of ship radiated noise data. Features extracted by the deeper layer are more discriminative than those from the shallow layer.

## 5. Conclusions

A deep convolutional neural network with auditory-like mechanisms is proposed to simulate the processing procedure of an auditory system for ship type classification. The integrated auditory mechanisms from early to higher auditory stages include auditory filters at basement membrane, neural pattern transformation by hair cells, spectro-temporal patterns along hierarchical structure, multiple auditory pathways and plasticity.

The classification experiments demonstrate that the proposed method outperforms manually designed features and classifiers. This study analyzes the recognition results in a way that is closer to the real-world scenario. The accuracy of recordings obtained by majority voting is much higher than the accuracy of segments. The increase of distance between ships to hydrophone has a negative effect on the recognition results in most cases. The proposed method has robustness to ship operative conditions. The network could generate a spectrogram that is similar to gammatone spectrogram, but smooth noises of high frequency components. The auditory filter banks in the network are adaptive in shape to ship radiated noise.

The proposed method facilitates the development of a smart hydrophone that could not only measure underwater acoustic signals, but also send alerts if it detects a specific underwater acoustic event. It will make it easier for researchers to listen to the ocean.

## Figures and Tables

**Table 1:** Comparison of the proposed model and other methods.

Input	Model	Accuracy (%)
Waveform [1,2]	SVM	68.2
MFCC [3]	BPNN	72.1
Wavelet,Waveform,MFCC,Auditory feature [7]	SVM Ensemble	75.1
Wavelet and principal component analysis [5]	BPNN	74.6
Spectral [11]	Stacked Autoencoder	81.4
Spectral [27]	CNN	83.2
Time domain	Auditory inspired CNN [6]	81.5
Time domain	Proposed	87.2

**Table 2:** Confusion matrix of samples.

	Predicted	Background	Cargo	Tanker	Passenger	Tug	Recall (%)
Ture							
Background		15,824	1	202	20	173	97.56
Cargo		16	13,152	2424	560	155	80.65
Tanker		120	1479	13,283	881	610	81.13
Passenger		133	356	233	14,908	748	91.02
Tug		334	317	590	1098	14,083	85.76
Precision (%)		96.33	85.93	79.39	85.35	89.31	87.2

**Table 3:** Confusion matrix of recordings.

	Predicted	Background	Cargo	Tanker	Passenger	Tug	Recall (%)
Ture							
Background		50	0	0	0	0	100
Cargo		0	107	9	2	0	90.68
Tanker		0	3	76	2	1	92.68
Passenger		0	0	1	137	3	97.16
Tug		0	0	0	2	56	94.92
Precision (%)		98.04	97.27	88.37	95.80	93.33	94.75
